# ESPO/SOCIAL RESEARCH, POLICY, AND PRACTICE: CHOT: AN INDUSTRY-ACADEMIC MODEL TO IMPROVE HEALTH

**DOI:** 10.1093/geroni/igz038.1545

**Published:** 2019-11-08

**Authors:** Justin C Lord, Danielle Waldron

**Affiliations:** 1 Louisiana State University at Shreveport, Shreveport, United States; 2 Department of Gerontology, University of Massachusetts Boston, Boston, Massachusetts, United States

## Abstract

This proposed symposium will bring together a diverse panel of emerging and established academics, as well as, industry leaders to speak about the power of collaboration. As the delivery of health care in the field of gerontology becomes increasingly complex – researchers and practitioners will have to find new ways to address these challenges. One potential solution is to become more interdisciplinary and collaborative in our approach. This symposium will bring together academic and industry leaders from the National Science Foundation’s Centers for Health Organization Transformation (CHOT) to share with our membership an industry-academic partnership model that has benefited industry-focused research across more than 50 disciplines. As the ESPO representatives for the SRPP division, we believe that our membership would benefit from a symposium of this nature. This symposium would also reflect this year’s conference is “Harnessing the Power of Networks.” The SRPP section has the strongest commitment to practice and research on practice such as ways to improve the delivery of services to an increasingly diverse older population. A model of industry partnership holds tremendous value for all those involved; however, it is incredibly valuable for junior researchers who are just beginning their careers. The CHOT model provides opportunities and funding for researchers to develop collaborative relationships; engage in research; access new sources of data; disseminate findings directly to a broader audience, and have the ability to address some of health care’s wicked problems.

